# Consumer Evaluation of the Quality of Online Health Information: Systematic Literature Review of Relevant Criteria and Indicators

**DOI:** 10.2196/12522

**Published:** 2019-05-02

**Authors:** Yalin Sun, Yan Zhang, Jacek Gwizdka, Ciaran B Trace

**Affiliations:** 1 School of Information The University of Texas at Austin Austin, TX United States

**Keywords:** health information quality, health information seeking, consumer health informatics, online health information

## Abstract

**Background:**

As the quality of online health information remains questionable, there is a pressing need to understand how consumers evaluate this information. Past reviews identified content-, source-, and individual-related factors that influence consumer judgment in this area. However, systematic knowledge concerning the evaluation process, that is, why and how these factors influence the evaluation behavior, is lacking.

**Objective:**

This review aims (1) to identify criteria (rules that reflect notions of value and worth) that consumers use to evaluate the quality of online health information and the indicators (properties of information objects to which criteria are applied to form judgments) they use to support the evaluation in order to achieve a better understanding of the process of information quality evaluation and (2) to explicate the relationship between indicators and criteria to provide clear guidelines for designers of consumer health information systems.

**Methods:**

A systematic literature search was performed in seven digital reference databases including Medicine, Psychology, Communication, and Library and Information Science to identify empirical studies that report how consumers directly and explicitly describe their evaluation of online health information quality. Thirty-seven articles met the inclusion criteria. A qualitative content analysis was performed to identify quality evaluation criteria, indicators, and their relationships.

**Results:**

We identified 25 criteria and 165 indicators. The most widely reported criteria used by consumers were trustworthiness, expertise, and objectivity. The indicators were related to source, content, and design. Among them, 114 were positive indicators (entailing positive quality judgments), 35 were negative indicators (entailing negative judgments), and 16 indicators had both positive and negative quality influence, depending on contextual factors (eg, source and individual differences) and criteria applied. The most widely reported indicators were site owners/sponsors; consensus among multiple sources; characteristics of writing and language; advertisements; content authorship; and interface design.

**Conclusions:**

Consumer evaluation of online health information is a complex cost-benefit analysis process that involves the use of a wide range of criteria and a much wider range of quality indicators. There are commonalities in the use of criteria across user groups and source types, but the differences are hard to ignore. Evidently, consumers’ health information evaluation can be characterized as highly subjective and contextualized, and sometimes, misinformed. These findings invite more research into how different user groups evaluate different types of online sources and a personalized approach to educate users about evaluating online health information quality.

## Introduction

More than 70% of US adults search online for health information [1]. The information found online shapes and influences consumers’ health beliefs, intentions, health behaviors, and health care decision making [2-5]. Since the inception of the internet, the quality of health information has been a source of concern for stakeholders due to the unregulated nature of the medium [6]. This concern is furthered by the fast growth of social media and user-generated content and corroborated by more than 200 evaluation studies conducted by subject experts, which collectively suggest that the quality of consumer-oriented health information on the internet varies greatly and that the overall quality was low [7] and remains low [8].

Making decisions based on low-quality health information (eg, information that is inaccurate, incomplete, or biased) may lead to harmful consequences, such as delayed treatment or extreme anxiety [9], and subsequently increase consumer vulnerability [10,11]. Nevertheless, evaluating the quality of information has been a major challenge for online health consumers [12-14]. For example, some consumers are uncertain about the accuracy, completeness, and validity of the information they encounter [15,16]; some cannot differentiate between scientific facts, empirical factors, and personal opinions [17]; and others suffer from information overload and subsequently lack the confidence and ability to evaluate information [18-21]. Studies have found that compared to health care providers or information professionals, consumers tend to give higher quality ratings to health information from both traditional health websites [22] and social media sites [23].

The ability to critically evaluate the quality of health information is an important component of health literacy [10], which is an important determinant of health [24]. To enhance this ability (and related skills), it is necessary to understand how consumers evaluate the quality of health information in the current internet environment. Consumer evaluation is subjective, driven by one’s information needs. Therefore, as a starting point, we adopted a broad conceptualization that defines quality through “fitness for use” [25]: Information is of good quality when it serves users’ needs. It is worth noting that this concept of quality is described using different terms in the existing literature, including but not limited to quality, credibility, trust, reliability, believability, and usefulness. In this review, we included articles using all these terms. We chose to be inclusive, because we want to achieve a comprehensive view of the assessments that consumers perform in the process of determining whether they would be willing to use a piece of information.

Guided by this understanding of quality, three recent systematic reviews were identified as relevant to our current research: One review focused on identifying factors that impact consumer judgment of trustworthiness and credibility of online health information [11], the second one identified the antecedents of trust in health information websites [27], and the final one reviewed the association between low health literacy and perceived quality and trust in online health information and low literacy consumers’ ability to evaluate information quality [10]. These reviews revealed that consumers’ quality evaluation is influenced by both source- and content-related factors [10,11,27]. Examples of source-related factors are website design (eg, layout, visual design, and interactive features), loading speed, and the authority of the owner or sponsor [11,27-30]. Examples of content-related factors are the authority of the author, content readability, content organization, use of evidence and citations, and the appearance of advertisements [11,27,31-33]. Additionally, a number of individual characteristics were identified as influencers, including demographics (eg, age, gender, and educational attainment), perceived health status, knowledge about the content, health beliefs, and level of health literacy [10,11,22,27,31].

These reviews provide an informative overview of factors that influence consumer online health information evaluation behavior but shed limited light on why and how these factors influence the evaluation behavior. From the perspective of information seeking, evaluation of information is a judgment and decision-making process that precedes users’ acceptance or rejection of received information [34]. Judgment and decision making involve applying certain criteria, principles, or standards to form evaluations [35]. Thus, to understand consumer quality evaluation behavior, it is necessary to understand the criteria used to guide the evaluation. Among the previously mentioned systematic reviews, only one [10] summarized the evaluation criteria reported in five studies on consumers with low health literacy. A more comprehensive understanding of the evaluation criteria is needed. This review intends to fill this gap.

Evaluation of the quality of online health information is a process of applying criteria to evaluate information. Thus, in addition to applying criteria, we need a better understanding of how consumers perceive online information. To achieve this goal, we deliberately differentiate between two concepts: criteria and indicators. Criteria are rules or filters that people apply to an information object to assess its value or worth [36]. Indicators, also termed cues or markers [37], are perceivable elements associated with an information object that allow people to reflect on the quality of the object [8]. Criteria are abstract, reflecting one’s values and preferences and mediating information selection decisions. Indicators are affordances of information objects that trigger or support the application of the criteria. Criteria are comparatively stable, whereas indicators are amenable to change. New indicators could emerge, and old ones could disappear with the development of new technologies and design preferences.

In this article, we focus on the following research questions: (1) What criteria do consumers use to evaluate the quality of online health information? (2) What elements of information objects do consumers use as quality indicators? (3) Which indicators convey positive evaluations and which convey negative evaluations? (4) What is the relationship between indicators and criteria, that is, what criteria do each indicator correspond to? We argue that a more comprehensive understanding of criteria used in the evaluation process can bring some clarity to the dimensions of quality perceived by online health consumers as well as their quality evaluation process. By explicating the relationship between indicators and criteria and identifying positive and negative judgments that indicators convey, the results can also inform the design of more user-friendly health information content and information systems.

## Methods

### Search Strategies

Seven online databases, including PubMed, Web of Science, PsycINFO, CINAHL (Cumulative Index to Nursing and Allied Health), Cochrane Library, Library and Information Science Source, and Communication and Mass Media Complete, were searched in July 2017 to obtain relevant journal articles. These databases were chosen because they cover major academic disciplines that study consumer online health information search, including health, information and library science, psychology, and mass communication. Keywords, including *quality*, *credibility*, *trust*, *reliability*, *accuracy*, *readability*, *relevance*, and *usefulness* were used in combination with the keywords *consumer or patient* and *online health information evaluation or online health information assessment*. After the searches, we manually screened the references to identify relevant articles and further examined the reference lists of these articles. Additionally, we examined the references cited in the three systematic reviews mentioned above and articles that cited these reviews (using Google Scholar’s “cited by” function).

### Inclusion/Exclusion Criteria

Articles meeting the following criteria were eligible for inclusion in this review: (1) The study primarily focused on consumer evaluation of health information on the internet. Health consumers include patients, caregivers, and the general public who sought or were interested in seeking health information. This focus differentiates this review from prior reviews of health care professionals or expert evaluation of online health information for consumers [7,8]. Articles that focus on media other than the internet (eg, TV and radio) were excluded. (2) The study was empirical and based on direct inquiries with health consumers where criteria were described by participants and not imposed by researchers. Articles that used only predefined evaluation criteria to survey consumers or analyze their responses without allowing new criteria to emerge were excluded. We also excluded correctional studies that focus on identifying factors (eg, source expertise) influencing consumer evaluation behavior but do not provide additional results on how quality evaluation is performed. (3) The article was published after 2002, when research on consumer evaluation of online health information began to emerge. (4) The article was written in the English language. (5) The article was published in a peer-reviewed journal.

### Study Identification

**Figure 1:**
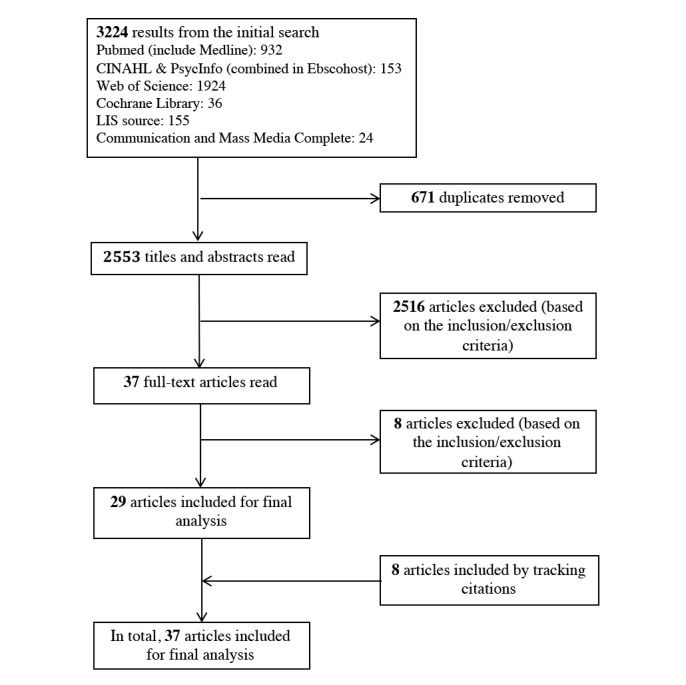
Article screening process.

Figure 1 shows the process involved in identifying eligible studies. Three authors (YS, YZ, and JG) reviewed a subset of the search results by reading titles and abstracts. YS and YZ both reviewed 10% of the records (256 records in total) to check the intercoder agreement in filtering potentially relevant articles (Cohen kappa=0.83). Both YS and YZ screened the full-text articles. When there was uncertainty involved in excluding a full-text article, the other two authors provided their input.

### Data Extraction and Analysis

Full text of the 37 selected articles was imported into MAXQDA 12 (VERBI Software GmbH, Berlin, Germany) for analysis. We extracted the following information: basic characteristics of the articles (eg, year of publication, country of origin, health topics, and aims of the study), research methods, sampling techniques, participant characteristics (eg, demographics and disease experiences), source studied (eg, the internet or specific health websites), and characteristics of the search tasks (eg, self-generated vs assigned) when search tasks were involved. Guided by their corresponding definitions, indicators and the corresponding criteria were extracted from the results and discussion reported in the original papers. When no clear relationships were reported (in most of such cases, indicators were reported without mentioning the criteria. For example, .com was reported as a negative indicator of quality, but criteria by which this judgment was reached were not reported), the authors of the review derived the relationships from the participants’ direct quotes reported in the original papers, the original authors’ discussion of the results, or the interpretation of the authors of the review. Indicators were further coded into positive (+, entailing positive quality judgment), negative (–, entailing negative judgment), or both (±, entailing both positive and negative judgments). When participants commented on the absence of an indicator (eg, no author credential or no advertisements), it was coded as positive if the absence implies low quality and as negative if the absence implies high quality. The criteria were also coded into the three categories based on their correspondence with indicators.

We analyzed the basic characteristics of the included studies using descriptive statistics. The qualitative content analysis method [26] was used to identify themes and build categories based on the extracted information concerning criteria and indicators in an iterative manner. YS coded all the articles. YZ validated the results by comparing each assigned code to the full-text of the articles. A number of group meetings were held to discuss the codes, especially relationships between indicators and criteria. Discrepancies were discussed among all authors.

## Results

### Basic Characteristics of the Included Articles

The 37 articles included in the review were published between 2002 and 2017. They originated from 8 countries, primarily United Kingdom (N=12), United States (N=11), Australia (N=4), and the Netherlands (N=3). The characteristics of each included article are summarized in Table 1.

Focus groups (n=17), interviews (n=16), and observations of participants performing predefined (n=11) or self-generated (n=6) search tasks were the primary research methods used in the selected articles. Observations were often used with other methods including think aloud, guided interviews, focus groups, or diaries. Fourteen articles used multiple research methods.

Twenty-one articles focused on information on a specific health condition or issue (eg, HIV prevention, diabetes, disabilities, and chronic diseases), and the remaining articles did not specify a subject focus. Twelve studies recruited patients with a specific condition, and the others recruited people who had searched online for health information (n=6) or had a strong interest in their health or a particular condition (n=5). Twenty-eight articles involved adult participants (≥18 years old), of which 10 articles also involved older adults (>64 years old). Four studies included adolescents aged 11-17 years. The number of participants ranged from 5 to 188 (median=21). In terms of sampling technique, 26 articles used purposive sampling, five used convenience sampling, and the remaining six did not report the sampling methods.

**Table 1:** Characteristics of the included articles.

Articles	Health topics	Source studied	Sampling method	Participants			Data collection methods
				N	Age range (years)	Disease experience	
Eysenbach and Köhler [30]	Not specified	The internet, in general	Purposive	21	19-71 (mean=37)	Healthy volunteers who had searched online for health information	Focus groups, naturalistic observation of consumers searching predefined search tasks, and follow-up interviews
Frisby et al [39]	Smoking cessation	The internet, in general	Convenience	13	19-64	Smokers from a smoking cessation campaign	Interviews, observations of participants searching both predefined and self-selected search tasks, and think aloud
Peterson et al [40]	Medicines/drugs	The internet, in general	Purposive	46	18-67 (mean=41.7)	People who had searched online for health information	Focus groups
Williams et al [41]	Not specified	The internet, in general	Purposive	42	30-49	People who had searched online for health information	Open-question survey
Bernhardt and Felter [42]	Pre- and postnatal health	The internet, in general	Purposive	20	22-42 (mean=34.5)	Mothers of young children	Focus groups
Childs [43]	Not specified	The internet, in general	Not reported	35	Not reported	Parents and caregivers of children with rare diseases	Focus groups
Adam et al [44]	Not specified	The internet, in general	Purposive	18	20-60	People who had searched online for health information	Observation of participants searching both self-generated and predefined tasks, and semistructured qualitative interviews
Crystal and Greenberg [45]	Not specified	The internet, in general	Purposive	12	Not reported	People who have a strong interest in their health	Observation of participants searching self-generated search tasks, think aloud, and guided interviews
Kerr et al [46]	Chronic conditions (eg, Alzheimer disease)	Interactive health communication application	Purposive	40	30-79	Chronic disease patients and caregivers	Focus groups
Marshall and Williams [18]	Not specified	Preselected websites	Purposive	32	Not reported	Patients with various conditions and care givers	Information review groups
Hoffman-Goet and Friedman [47]	Breast cancer information	Preselected websites	Convenience	25	50-71 (mean=59.2)	Canadian aboriginal senior women	Interview
Sillence and Briggs [48]	Not specified	The internet, in general	Not reported	42	22-68	Internet users interested in their health	Focus groups
Sillence et al [32]	Menopause	The internet, in general, and preselected websites	Not reported	15	41-60 (mean=49)	Women faced with decisions concerning menopause and hormone replacement therapy	Observation of participants searching predefined and self-generated search tasks with think aloud and guided focus groups, and free search with diary keeping
Sillence et al [49]	Hypertension	The internet, in general, and preselected websites	Not reported	13	33-68	Hypertension patients	Observation of participants searching self-selected and predefined search tasks, with think aloud and guided focus groups, and free search with diary keeping
Buhi et al [50]	Sexual health	The internet, in general	Purposive	24	Not reported	First-year undergraduate students	Observation of participants searching predefined search tasks and think aloud
Freeman and Spyridakis [51]	Diabetes	The CDC^a^ website	Convenience	188	Mean=21	University students	Controlled experiment with open-ended questions in a questionnaire
Mackert et al [52]	Childhood obesity and nutrition	The internet, in general	Purposive	43	≥18	Parents with low health literacy	Focus groups
Marton [53]	Mental health	The internet, in general	Convenience	5	Not reported	Chronic mental health patients	Interviews
Kim et al [54]	Preconception nutrition	The internet, in general	Purposive	11	20-22	University students	Observation of participants searching predefined search tasks with guided interviews
Feufel and Stahl [13]	Not specified	The internet, in general	Purposive	22	>50 or <30 (mean for older cohort=65, mean for younger cohort=23)	Older vs younger cohorts (with different health literacy skills)	Observation of participants searching predefined search tasks and concurrent talk-aloud
Henderson and Eccleston [38]	Pain problem	The internet, in general	Purposive	13	12-17 (mean=14.38)	Adolescent users of online content for pain	Online focus groups
Colombo et al [55]	Multiple sclerosis	The internet, in general	Purposive	60	18-60	Multiple sclerosis patients and their family members	Offline/online focus groups
Lederman et al [56]	Not specified	Online forums	Purposive	16	≥18	Consumers who had searched online for health information	Interviews
McPherson et al [57]	Chronic conditions	Preselected websites	Purposive	6	11-23 (mean=16.7)	Children and young people with chronic conditions	Focus groups
Payton, et al [58]	HIV prevention	The NIH^b^ website	Not reported	40	18-24	Black female college students	Focus groups
Briones [59]	Not specified	The internet, in general	Purposive	50	18-25	University students	Interviews
Rennis et al [60]	Not specified	The internet, in general	Convenience	14	Mean=25.71	Urban community college students	Focus groups
Santer et al [20]	Childhood eczema	The internet, in general	Purposive	28	26-46 (median=36)	Parents of children with eczema	Interviews
Subramaniam et al [61]	Obesity and other general health issues	The internet, in general, and preselected obesity websites	Purposive	30	10-15 (mean=12.8)	Adolescents from low socioeconomic status and minority family	Participants searching self-selected health topics followed by search log analysis, and interviews of preselected websites
Cunningham and Johnson [62]	Not specified	Patients.co.uk	Not reported	11	Not reported	General public	Observation of participants searching predefined search tasks and concurrent talk-aloud
Diviani et al [63]	Not specified	The internet, in general	Purposive	44	Mean=37	Italian-speaking adults with different health literacy levels	Interviews
Sillence et al [64]	“Raw” milk	Pre-selected raw milk websites	Purposive	41	24-85 (mean=48)	Milk consumers	Observation of participants searching predefined search tasks, log analysis, and guided group discussion
Alsem et al [65]	Physical disabilities	The internet, in general	Purposive	15	26-58	Parents of children with physical disabilities	Interviews
Champlin et al [66]	Not specified	The internet, in general	Purposive	40	Mean=39	People with different health literacy levels	Interviews
Cusack et al [67]	Not specified	The internet, in general	Purposive	27	12-15	Students in grades 7-9	Interviews
Peddie and Kelly-Campbell [68]	Hearing health	The internet, in general	Purposive	11	44-84 (median=70)	Hearing-impaired patients	Observation of participants searching predefined search tasks, think aloud, and guided interviews
Scantlebury et al [69]	Not specified	The internet, in general	Purposive	14	21-70	People who had searched online for health information	Focus groups

^a^CDC: Centers for Disease Control and Prevention.

^b^NIH: National Institutes of Health.

Regarding evaluation of internet sources, 28 articles did not specify a scope. The remaining nine articles specified or preselected sources for evaluation (eg, pediatric sun protection websites, the National Institutes of Health website, the Centers for Disease Control and Prevention website, patients.co.uk, and online forums).

### Quality Evaluation Criteria Used by Consumers

Twenty-five criteria were identified (Table 2). The definitions were derived from the codes or drawn directly from the included studies.

Among these criteria, trustworthiness, expertise, and objectivity were reported most often in the articles, followed by transparency, popularity, and understandability. Eight criteria including relevance, familiarity, accessibility, identification, believability, accuracy, readability, and currency were reported in 10-15 articles. The remaining 11 criteria appeared in less than 10 articles.

### Quality Indicators Used by Consumers

Indicators used by consumers to evaluate the quality of online health information were related to three aspects of online information: source, content, and design. Table 3 shows their distribution across the three categories.

About 52% of the indicators were content related, followed by design (25%) and source factors (23%); 69% of the indicators were associated with positive quality judgment, 21% were associated with negative quality judgment, and 10% could lead to both positive and negative judgment.

**Table 2:** Criteria used by consumers to evaluate the quality of online health information.

Criterion	Definition	Articles reporting the criterion, n (%)
Trustworthiness	Whether a source or information is honest or truthful and can be trusted	31 (84)
Expertise	Whether a source or author has a sufficient level of subject-related knowledge	31 (84)
Objectivity	Whether a source or information presents facts that are not influenced by personal feelings or commercial interests	30 (81)
Transparency	Whether important information that influences a user’s ability to make informed choices (eg, motivation of a site or owner contact information) are disclosed	21 (57)
Popularity	Whether a source or information appears in multiple venues or is received or accepted by a large number of people (eg, ranked high in search engines or followed or accepted by the crowd in social media)	19 (51)
Understandability	Whether a source or information is in appropriate depth, quantity, and specificity and error free	18 (49)
Relevance	Whether information is relevant to the topic of interest or to information seekers’ situation and background	15 (41)
Familiarity	How familiar the source is to an individual	14 (38)
Accessibility	Whether a source is easy to access and stable	14 (38)
Identification	Whether a source or information conforms to an individual’s identity, goals, styles, arguments, or objectives [62].	13 (35)
Believability	Whether information is logical and can be believed	12 (32)
Accuracy	Whether a source or information is consistent with agreed-upon scientific findings	12 (32)
Readability	Whether information is presented in a form that is easy to read (eg, concise and clear layout)	10 (27)
Currency	Whether a source or information is up to date	10 (27)
Navigability	Whether a source or information is organized in a way that is easy to navigate	9 (24)
Aesthetics	Whether the appearance of the interface is visually pleasing	9 (24)
Interactivity	Whether a source offers sufficient functions to allow users to interact with the source	9 (24)
Comprehensiveness	Whether a source or information covers a wide range of topics or offers different interaction features (eg, shopping, socializing, and researching)	8 (22)
Practicality	Whether information can be readily applied by an individual (eg, personal advice and experience)	8 (22)
Completeness	Whether necessary or expected aspects of a subject/topic are provided	7 (19)
Usefulness	Whether the amount, depth, or specificity of a source or information are at an appropriate level that can be used by an individual	7 (19)
Balanced	Whether different perspectives concerning a topic or both pros and cons concerning a treatment are provided	6 (16)
Anonymity	Whether a source can be used without forcing users to provide personal information	3 (8)
Security	Whether a source is able to prevent malicious attacks (eg, virus)	2 (5)
Learnability	Whether information can satisfy different learning needs (eg, people with different levels of knowledge)	2 (5)

**Table 3:** Distribution of quality indicators used by consumers to evaluate the quality of online health.

Indicators	Positive, n (%)	Negative, n (%)	Positive and negative, n (%)	Total, n (%)
Source	24 (63)	5 (13)	9 (24)	38 (23)
Content	62 (73)	17 (20)	6 (7)	85 (52)
Design	28 (67)	13 (31)	1 (2)	42 (25)
Total	114 (69)	35 (21)	16 (10)	165 (100)

### Source

Source is the entity that creates, hosts, or distributes content. A source can be a website or the owner, creator, or sponsor of the site. Six categories of source-related quality indicators were identified: site owners/sponsors, site types, disclosures, third-party accreditations, recommendations from other systems or users, and website scope. More detailed indicators reported in the included articles, their direction of influence on quality judgment (positive, negative, or both), the corresponding criteria that guide the consumers’ appraisal of the indicators, as well as the value of the criteria (positive, negative, or both) are shown in Table 4. The indicators in the tables are self-explanatory; therefore, we focus on describing the most frequently appearing indicators in the included studies and indicators that can lead to both positive and negative judgments.

#### The Most Frequently Mentioned Indicators

The most frequently mentioned source-related indictors were site owners/sponsors, with sites run by reputable organizations, educational and academic institutions [18,40,41,46, 52,59], and medical experts and health institutions [32,39,44,46,51, 54,55,57,59,65] being considered more trustworthy and offering higher levels of expertise. The second most frequently reported indicators were about disclosure. Sites that disclose their motivations were highly valued [40,42,48,64,66,67], whereas a lack of a clear statement of purpose and motivation damaged trust [49]. The third mostly frequently reported indicators were recommendations from other systems or users. High ranks in search engines [13,52,63] and a large number of visitors or followers [61,63] were viewed as indicators of high site popularity, and subsequently, high quality. In addition, sites linked from or recommended by a trusted website [30,43,53] or trusted others (eg, health care providers, families, and friends) [20,41,47,61,67] were considered trustworthy.

#### Indicators With Both Positive and Negative Influences on Evaluation

Mixed attitudes were found toward some indicators representing site owners/sponsors. First, most participants believed that government websites (eg, National Health Service and Centers for Disease Control and Prevention) reflect high levels of expertise and good intentions [39-41,46,53,54,58,59,64,65]; however, some consumers suspected that the information on government websites is biased due to their agendas [41,52], and some, particularly younger generations, did not identify themselves with government sources, considering them “less cool” and not relatable [58]. Second, most people considered sites operated by local health societies to have a high level of expertise; however, some minorities and people from nonmainstream cultures (eg, aboriginal communities) were likely to question the relevance and accuracy of the information from these sites [47]. Third, people usually considered websites owned by commercial companies less objective [42,46,48] and trusted more websites with no commercial interests [18,20,42,55,67]; nevertheless, popular commercial websites such as BabyCenter.com, ParentsPlace.com, and WebMD.com were favored by some people for their expertise and comprehensiveness [42]. Fourth, a few people viewed information from pharmaceutical company websites as “official” [40], whereas others considered their information biased due to the financial interests involved [32,40,46,48].

Consumers had mixed attitudes toward the website types, particularly social media sites. Some consumers favored online discussion groups, chat rooms, and listservs because they offered first-person narratives and practical information and support from peers with whom they could identify (ie, those who have similar conditions) [46,53,57], but some disliked such sites for their lack of objectivity and expertise [13,53,59]. Concerning Wikipedia, some people questioned its objectivity because information can be edited by anyone on the Web [50,58,61], but some consumers were attracted to its encyclopedic nature and comprehensiveness [63].

Consumers also had different opinions regarding sites recommended by others. Some trusted a site recommended by trusted others (eg, health care providers, families, and friends) [20,41,47,61,67]; however, some consumers recognized that recommendations from other individuals may not be relevant to their situation [67].

**Table 4:** Evaluation of the source.

Indicators			Criteria
**Site owners/sponsors (n=30)**			
	**Site name (n=4) [32,40,63,64]**			
		Inappropriate or weird site names (–^a^)	Believability (–)
	**Domain type (n=5)[40,42,50,57,61]**		
		.com (–)	Objectivity (–)
		.org (+^b^)	Trustworthiness (+)
		.gov (±^c^)	Expertise (+), Trustworthiness (±)
		.edu (+)	Expertise (+), Trustworthiness (+)
	**Owner identity (n=26)[18,20,30,32,32,39-42,44,46,48-55,57-59,64,65,67,68]**		
		Individual sponsor (–)	Objectivity (–)
		Private sites (–)	Objectivity (–)
		Reputable organizations (+)	Trustworthiness (+)
		Educational and academic institutions (+)	Expertise (+)
		Medical or health institutions/experts (+)	Expertise (+)
		Scientific publisher (+)	Expertise (+)
		Patients’ organization (+)	Trustworthiness (+)
		Well-known news sites (+)	Trustworthiness (+)
		Government institutions (±)	Expertise (+), Trustworthiness (±), Identification (–)
		Local cancer society (±)	Expertise (+), Relevance (–), Accuracy (–)
		Commercial sponsor (±)	Objectivity (–), Expertise (+)
		No financial gain to the owner (+)	Objectivity (+)
		Pharmaceutical industry (±)	Expertise (+), Objectivity (–)
**Site types (n=9) [13,46,50,53,57-59,61,63]**			
	Online peer support and discussion groups (+)		Identification (+), Practicality (+)
	Chatrooms (+)		Identification (+), Practicality (+)
	Forums (–)		Objectivity (–), Expertise (–)
	Personal blogs/websites (±)		Objectivity (–), Expertise (–), Identification (+)
	Listservs (±)		Objectivity (–), Expertise (–), Identification (+)
	Wikipedia (±)		Objectivity (–), Expertise (–), Comprehensiveness (+)
**Disclosure (n=13) [30,40-43,48,49,51,55,63,64,66,67]**			
	Disclosure of the site owner (+)		Transparency (+)
	Age of a website (+)		Transparency (+), Trustworthiness (+)
	Picture of the site owner (+)		Transparency (+)
	Contact information (+)		Transparency (+)
	Motivation of the site (+)		Transparency (+)
	Explicit disclaimer and alert (+)		Transparency (+)
**Third party accreditation (n=4) [30,43,46,63]**			
	Quality certificates, seals, stamps, or kitemarking (+)		Accuracy (+)
**Recommendations from other systems or users (n=12) [13,20,30,41,43,47,52,53,61,63,67]**			
	Rank in search engine results (+)		Popularity (+)
	Number of site visitors or followers (+)		Popularity (+)
	Titles and excerpts in search engine results (+)		Relevance (+)
	Linked from a trustworthy site (+)		Trustworthiness (+)
	Recommended by other people (±)		Trustworthiness (+), Relevance (–)
**Website scope (n=7) [32,42,44,49,53,54,58]**			
	A wide range of topics in a site (+)		Comprehensiveness (+)
	Multiple functions in a site (+)		Comprehensiveness (+)

^a^– indicates a negative evaluation of quality or that a criterion is judged negatively.

^b^+ indicates a positive evaluation of quality or that a criterion is judged positively.

^c^± could indicate both positive and negative evaluations or a criterion could be judged both positively and negatively.

### Content

Content refers to the information contained in a source as well as the presentation of the information. Eight categories of content-related indicators were identified: substance, writing and language, presentation, references, authorship, audience, date/updating, and advertisements. Table 5 shows these indicators, the corresponding criteria that guide the consumers’ appraisal of the indicators, and their influence on quality judgment.

#### The Most Frequently Mentioned Indicators

The most frequently reported content indicators were about consensus among sources. Content that appears in multiple sources, be it online sources, sources in other media (eg, newspaper, television, books, and academic journals), or health care professionals, is trusted by consumers. Writing- and language-related factors were the second most frequently reported content indicators. Consumers expect high-quality information to be error free in spelling and grammar, use straightforward language, and have a clear layout. The third most frequently reported indicators were advertisements. Consumers expect quality websites to neither depend on advertisements [63] nor seek to make a profit [62]. Therefore, sites with advertisements were considered less objective [46,49,54,63,64], be it in the form of commercial links [46], advertisement banners [30,32,55], popups [32,40], or other formats.

#### Indicators With Both Positive and Negative Influences on Evaluation

Consumers had mixed attitudes toward two content types: objective facts and personal experiences. Some consumers value objective facts [43,45,62], whereas some dissatisfy with information that contained solely objective facts, considering it unbalanced [69]. With regard to personal experiences, some consumers favored first-hand experiences, stories, and advice (eg, recommendations for medical gadgets, meal planning and exercising, and advice on completing medical benefit forms) from people with similar conditions for their practicality [40,41,45,46,49,63,64], but some had concerns that personal experiences lack objectivity, especially when it merely is a personal opinion [30,69].

Celebrity endorsement was also an indicator with both positive and negative influences on quality evaluation. Some trusted the endorsed information but others question its objectivity due to the potential financial interest involved [61,67].

The use of medical and technical vocabularies raised contention among consumers as well. For some consumers, high-quality information was easy to understand, that is, it exhibited less use of professional medical vocabularies [30,43,49,64,69] or provided easy-to-understand definitions of medical jargon [46,49], especially for educational and government sites [18,42,52]; however, for others, the use of technical vocabularies demonstrated expertise and was highly valued [51,60,63].

Some consumers doubted information (especially diagnosis and treatment information) authored by other unknown consumers [42,53], but others tended to trust content written by their peers because of similar demographic or health characteristics [32].

For health interventions, some consumers examined the release time and perceived newer interventions to have higher quality than the existing ones because the new intervention would have undergone more testing and research, whereas others were uncertain about the reliability of newer interventions [67].

### Design

Design refers to the appearance of a website or an app and the interactions that it affords. Four categories of design-related quality indicators were identified: interface design, interaction design, navigation design, and security settings. Table 6 shows the specific indicators, the corresponding criteria that guide the consumers’ appraisal of the indicators, and their influence on quality judgment.

**Table 5:** Evaluation of content.

Indicators				Criteria
**Substance (n=31)**			
	**Content types (n=15)**			
		**Factual information (n=14) [18,30,40,41,43,45,46,49,53,62-64,67,69]**	
			Evidence based (+^a^)	Objectivity (+)
			Clinically proven (+)	Objectivity (+)
			Statistics and numbers (+)	Objectivity (+)
			Concrete examples (+)	Practicality (+)
			Objective facts (±^b^)	Objectivity (+), Balanced (–)
			Ideological and magical information (–^c^)	Accuracy (–)
			Unproven and uncertain scientific information (–)	Accuracy (–), Objectivity (–)
		**Personal experiences (n=9) [30,40,41,45,46,49,63]**	
			First hand (+)	Accuracy (+)
			Practical advice (+)	Practicality (+)
			Personal experiences (±)	Objectivity (–), Practicality (+), Identification (+)
			Personal opinion (–)	Objectivity (–), Expertise (–)
	**Content attributes (n=29)**			
		**Balance (n=6) [43,46,48,63,64,67]**		
			Alternative medicine (+)	Balanced (+)
			Conflicting views (+)	Balanced (+)
			Both professional and patient viewpoints (+)	Balanced (+)
			Potential side effects (+)	Complete (+), Transparency (+)
		**Depth (n=5) [18,46,49,51,62]**		
			At the right level of complexity and depth (+)	Understandability (+)
			Increasing in depth overtime (+)	Usefulness (+)
			In-depth information (+)	Expertise (+)
		**Quantity (n=5) [18,46,62,67]**		
			The right amount (+)	Understandability (+)
			Too much text (–)	Understandability (–)
		**Specificity (n=5) [18,46,47,49,67]**		
			Various levels of detail for different needs (+)	Usefulness (+)
			Specific and detailed (+)	Understandability (+)
			Overall and general information (–)	Usefulness (–)
		**Consensus among sources (n=20) [13,18,30,38-43,55-57,59-61,63,65-67,69]**		
			Reaching agreement among media sources (+)	Popularity (+)
			Verified by general practitioners or other health professionals (+)	Expertise (+)
			Crowd consensus (+)	Popularity (+)
			Endorsed by celebrities (±)	Trustworthiness (+), Objectivity (–)
	**Specific content elements (n=3) [47,61,67]**			
		Natural ingredients (+)		Trustworthiness (+)
		Amount of investment on an intervention (+)		Trustworthiness (+)
		Copyright information (+)		Trustworthiness (+)
		Local support and contact information (+)		Usefulness (+), Relevance (+)
	**Argument strength (n=6) [30,32,41,48,56,69]**			
		Reasonable (+)		Believability (+)
		Sound plausible and scientific (+)		Believability (+)
		Biased or misleading (–)		Objectivity (–)
**Writing and language (n=19) [18,30,32,42,43,46,48,49,51,52,56-58,60,62-64,67,69]**				
	Spelling and grammar errors (–)			Understandability (–), Expertise (–), Trustworthiness (–)
	Long sentences (–)			Readability (–)
	Professional writing (+)			Expertise (+)
	Concise (+)			Comprehensiveness (+), Readability (+)
	Use simple, plain, straightforward, and clear language (+)			Understandability (+)
	Familiar sounding and inclusive language (+)			Understandability (+), Identification (+)
	Sensational (–)			Objectivity (–)
	Patronizing tone (–)			Identification (–)
	Use of professional medical terms and technical vocabularies (±)			Understandability (–), Expertise (+)
	Easy reading level (–)			Expertise (–)
**Presentation of content (n=12)**				
	**Organization (n=10) [18,32,44,45,49,54,59,62,66,68]**			
		Clear layout and organization (+)		Readability (+)
		An overview of the information on a site (+)		Readability (+)
		Use of bolding and shading (+)		Readability (+)
		Bulleted points (+)		Readability (+)
		Headings (+)		Readability (+)
		Prioritizing content (+)		Understandability (+)
		Structure of scientific papers: general approaches and research design (+)		Expertise (+)
		Structure of scientific papers: presence of variables or factors (+)		Expertise (+)
		Structure of scientific papers: research purposes (+)		Expertise (+)
	**Labeling (n=2) [43,63]**			
		Presence of an informative title (+)		Understandability (+)
		Clearly marked personal experience (+)		Transparency (+)
**References (n=10) [30,39,43,45,56-58,63,64,69]**				
	Links to original documents (+)			Transparency (+)
	Number of references included (+)			Trustworthiness (+), Expertise (+)
	Reference to scientific publications (+)			Objectivity (+), Expertise (+)
	Reference to a credible person (+)			Trustworthiness (+), Expertise (+)
	Reference to a specific project or institution (+)			Transparency (+), Trustworthiness (+)
**Authorship (n=16) [30,32,40,42,45,60,62-64,66,67]**				
	Explicitly listing authors and author’s credentials (+)			Transparency (+)
	Reference to previous work or curriculum vitae (+)			Trustworthiness (+), Expertise (+)
	Picture of the author (+)			Trustworthiness (+), Transparency (+)
	Health professionals (+)			Expertise (+)
	Journalists (+)			Expertise (+)
	Consumers (±)			Practicality (+), Identification (+), Expertise (–), Objectivity (–)
	Economic gains for its authors (–)			Objectivity (–)
	Religious figures (–)			Objectivity (–)
**Audience (n=11) [32,45-48,51,53,57,58,61,63]**				
	Targeted to geographical location (+)			Relevance (+)
	Translated information (+)			Understandability (+), Accessibility (+)
	Tailored and personalized information (+)			Usefulness (+)
	Targeted to minority women (+)			Identification (+)
	Targeted to professions (+)			Relevance (+)
	Targeted to age group (+)			Relevance (+)
	Seeing a face that looked similar to theirs (+)			Identification (+)
	Written for the most educated audience (+)			Expertise (+)
	Aimed at younger children (–)			Relevance (–), Accuracy (–)
**Date/updating (n=12) [30,39,41,43,44,46,53,59,60,63,67,69]**				
	The appearance of publication date (+)			Transparency (+)
	Access all the latest research (+)			Currency (+), Completeness (+)
	New interventions (±)			Currency (+), Accuracy (–)
	Up to date (+)			Currency (+)
	Regular updating (+)			Transparency (+), Currency (+)
**Advertisements (n=17) [30,32,39,40,42,43,46,49,54,55,59,62-64,66-68]**				
	Presence of ads (–)			Objectivity (–)
	Pushing to sell something (–)			Objectivity (–)
	The appearance of commercial links (–)			Objectivity (–)

^a^+ indicates a positive evaluation of quality or that a criterion is judged positively.

^b^± indicates both positive and negative evaluations or a criterion could be judged both positively and negatively.

^c^– indicates a negative evaluation of quality or that a criterion is judged negatively.

**Table 6:** Evaluation of design.

Indicators				Criteria	
**Interface design (n=16)**				
	**Overall appearance (n=9) [30,32,39,44,46,49,59,62,64]**			
		Boring and bland design (–^a^)		Aesthetics (–)
		Commercial nature/feel (–)		Objectivity (–)
		Modern look (+^b^)		Aesthetics (+), Identification (+)
		Professional (+)		Expertise (+), Trustworthiness (+)
		High visual quality (+)		Trustworthiness (+), Aesthetics (+)
		Soft colors (+)		Aesthetics (+)
	**Graphics (n=9) [18,32,40,44,49,54,64,66,67]**			
		Too many graphics (–)		Aesthetics (–)
		Use of flash (–)		Aesthetics (–), Accessibility (–)
		Poor graphics (–)		Aesthetics (–)
		Inappropriate graphics (–)		Relevance (–), Trustworthiness (–)
		The existence of brand logo (+)		Trustworthiness (+)
		Relevant illustrations (+)		Relevance (+)
	**Font (n=5) [18,32,44,49,68]**			
		Large font size (+)		Accessibility (+)
		Font color low contrast (–)		Accessibility (–)
**Interaction design (n=14)**				
	**Links (n=4) [45,48,49,55]**			
		Link to other websites (+)		Trustworthiness (+), Interactivity (+)
		Plenty of links (+)		Interactivity (+)
		Broken links (–)		Accessibility (–), Trustworthiness (–)
		Easy access to further details and sources (+)		Accessibility (+)
		Downloadable PDF documents for bibliographies and laws (+)		Accessibility (+)
	**Interactive functions (n=7) [30,32,44,46,49,61,62]**			
		Search capabilities (+)		Interactivity (+)
		Places to interact and share with other site visitors (+)		Interactivity (+)
		“Ask experts” (+)		Interactivity (+), Expertise (+)
		Self-management and assessment tools (±^c^)		Usefulness (+), Accuracy (–), Objectivity (–)
	**Other interactive features (n=9) [30,32,40,46,48,61,63,68,69]**			
		Slow loading time (–)		Accessibility (–)
		Required login (–)		Accessibility (–), Anonymity (–)
		Absence of pop-ups (+)		Accessibility (+)
		Multimedia feature (+)		Interactivity (+), Learnability (+)
**Navigation design (n=9) [30,32,44,46,49,54,58,68,69]**				
	Relevant info on home page (+)			Navigability (+)
	Clear entry point (+)			Navigability (+), Accessibility (+)
	Easy return to home page (+)			Navigability (+)
	Navigation aids (+)			Navigability (+)
	Navigation links (+)			Navigability (+)
	Site map (+)			Navigability (+)
	Side tool bars (+)			Navigability (+)
	Different ordering structures (+)			Navigability (+)
	Clear indication when taken offsite (+)			Navigability (+), Transparency (+)
	Easy transition between two or more sites (+)			Navigability (+)
	“Back” button as the only way to exit (–)			Navigability (–), Accessibility (–)
	Heavily relied on dropdown menu (–)			Navigability (–)
	Continually sending users offsite (–)			Interactivity (–), Trustworthiness (–)
**Security settings (n=2) [39,57]**				
	Secure sites (+)			Security (+)
	Recognized by antivirus software (+)			Security (+)

^a^– indicates a negative evaluation of quality or that a criterion is judged negatively.

^b^+ indicates a positive evaluation of quality or that a criterion is judged positively.

^c^± could entail both positive and negative evaluations or a criterion could be judged both positively and negatively.

#### The Most Frequently Mentioned Indicators

The most frequently reported design indicators were related to interface design, mostly visual factors, including the overall appearance of a site, the graphics it includes, and font size. Interaction design features, including links, interactive functions, and other interactive features (eg, loading time and login requirement), were the second most frequently mentioned quality indicators. Sites with robust search capabilities (eg, easy to locate and diverse search entrance), offering useful tools (eg, self-management tools), and rendering smooth user-system interaction (eg, providing links to additional relevant sources and not having pop-ups) were perceived as high quality. Navigation-related indicators such as navigation aids and site maps were the third most frequently mentioned quality indicators.

#### Indicators With Both Positive and Negative Influences on Evaluation

Mixed opinions existed concerning the interactive functions of self-management and assessment tools (eg, health calculators). Some consumers valued tailored results and advice [46,49], but some questioned the accuracy and objectivity of the information generated [46,62].

### Individual Factors Influencing Quality Judgment

In addition to source-, content-, and design-related factors, the evaluation of online health information quality was also affected by individual factors including individuals’ personal situation, prior knowledge or experience of a source, personal knowledge and beliefs, and intuition. Table 7 shows the specific factors, the corresponding criteria that guide the consumers’ appraisal, and their influence on quality judgment.

#### The Most Frequently Mentioned Factors

Individuals’ prior knowledge and experience of a source were mentioned most frequently as factors that influence quality judgment. Consumers tended to trust sites that they had experience with [49,63], because they may already know the source to be credible [13,18,39,50,53,59,65], have had positive experiences with it [42,54,67], have seen it from advertisements on other media (eg, television and magazine) [42,58], or are familiar with the organization behind the source [18,42].

The category of personal situation was the second most frequent factor. Information relevant to individuals’ search topics (eg, hormone replacement therapy) [32,45], needs and goals (eg, offering easy reading level message for younger people) [40,54,57], specific circumstances (eg, localization) [40,45,62,64], and experiences and symptoms [18,56,62,64] was considered to be of high quality.

The other two categories of individual factors were mentioned with the same frequency. One category is personal knowledge and beliefs. Consumers highly valued information consistent with their own beliefs and knowledge [18,32,41,56,63,64]. The other category is intuition. Some consumers undertook “subconscious filtering” to filter out potential political and gender biased information [69], and some consumers relied on common sense [39], sensation [63], instinct, or “gut feelings” [55,65,66] to evaluate information.

**Table 7:** Individual factors.

Factors		Criteria
**Individuals’ personal situation (n=9)** **[18,32,40,45,54,56,57,62,64]**		
	Relevant topics (+^a^)	Relevance (+)
	Information relevant to one’s needs and search goal (+)	Relevance (+)
	Information relevant to one’s circumstance and applicable (+)	Relevance (+) Usefulness (+)
	Information related to one’s experiences and symptoms (+)	Identification (+)
**Prior knowledge and experience of a source (n=14) [13,18,39,42,49,50,53,54,58,59,61,63,65,67]**		
	Known credible websites (+)	Familiarity (+) Expertise (+)
	Positive previous experience (+)	Familiarity (+) Trustworthiness (+)
	Websites advertised in other media (+)	Familiarity (+)
	Familiar organization (+)	Familiarity (+)
**Personal knowledge and beliefs (n=7) [13,18,32,41,56,63,64]**		
	Consistency with one’s own beliefs and knowledge (+)	Identification (+)
**Intuition (n=7) [39,55,57,63,65,66,69]**		
	Subconscious (+)	Believability (+)
	Common sense (+)	Believability (+)
	Instinct/sensation/gut feeling (+)	Believability (+)

^a^+ indicates a positive evaluation of quality or that a criterion is judged positively.

## Discussion

In this article, we reviewed 37 empirical studies that reported consumers’ accounts of how they evaluate the quality of online health information. This review extends the existing literature by making two major conceptual contributions. First, it offers a clear conceptual understanding of the dimensions of quality of online health information perceived by consumers by differentiating criteria from indicators. Second, it explicates the relationship between webpage quality indicators (webpage elements) and the quality judgment by differentiating positive and negative influences that indicators have on judgment. In this section, we discuss each contribution and then outline practical implications and limitations of this review.

### Dimensions of Online Health Information Quality

In the existing literature, quality was often defined and assessed differently. We guided the article selection for the review using a general conceptualization that defines quality as “fitness for use” [25]. Other authors have offered more specific conceptualizations. For example, Rieh [70] assessed quality as the extent to which users think that the information is useful, good, current, and accurate. Bates et al [71] measured health information quality in terms of its trustworthiness, truthfulness, readability, and completeness. Benotsch et al [22] rated the quality of health websites on five dimensions: accuracy, amount of detail, trustworthiness-credibility, relevance, and usefulness. Eastin [72] rated the credibility of health information on three dimensions: accuracy, believability, and factualness. The lack of consistency in measuring online health information quality suggests that there is a lack of clear conceptual understanding of what information quality means to online health consumers.

By clearly differentiating quality judgment criteria (rules that reflect notions of value and worth) and indicators (properties of information objects to which criteria are applied to form judgments) reported in the included studies, this review identified 25 dimensions (criteria) along which consumers evaluate the quality of online health information (Table 2). Because the included articles differ on aspects such as health issues of concern, participant demographics, and sources examined, this wide range of criteria reported and the uneven distribution of the criteria across the included articles suggest that consumer evaluation of online health information may be influenced by contextual factors such as user characteristics, health conditions, and online sources. In addition to these factors, the current review, consistent with prior reviews [10,11,27], also identified a range of individual factors that influence quality judgment behavior, such as prior experience with a source and personal knowledge and beliefs. Therefore, future studies should attempt to identify the most influential contextual factors (including individual factors) that affect consumers’ application of quality criteria to further enhance the theoretical understanding of this behavior. Empirical studies of consumer online health information evaluation should also consider these contextual factors in research design.

Despite the wide range, however, three criteria (trustworthiness, expertise, and objectivity) were reported in 31 articles, indicating that they are used consistently across user groups, source types, and health conditions and that they constitute core dimensions of online health information quality as perceived by consumers. The fact that trustworthiness and expertise are primary dimensions is consistent with general media source credibility research [73]. It is not surprising that objectivity, that is, whether a source or information presents objective factual or evidence-based information, is also important for health information. Three additional criteria—transparency (reported in 21 articles), popularity (reported in 19 articles), and understandability (reported in 18 articles)—are also commonly reported and could be viewed as secondary dimensions of online health information quality. These findings imply that consumers’ perceived online health information quality could be reasonably measured by a small set of core dimensions.

### Relationship Between Quality Indicators and Quality Judgment

Previous reviews summarized indicators used by consumers to evaluate the quality of online information [10,37]. Sbaffi and Rowley [11] further reported the direction of the effect (ie, positive vs negative) of the (design and content) indicators. However, the situational nature of the relationship between indicators and quality judgment, that is, the fact that their relationship is not one-on-one, but dependent on users’ values and the criteria applied, was not explicitly discussed. For example, government institutions, usually associated with high level of expertise and authority, are perceived by some consumers as biased sources with which they have a hard time relating. The other example is that consumer-generated content (eg, personal blogs and listserves) indicates low objectivity and low level of expertise to some consumers, but to others, it is considered highly practical and relatable. Thus, a unique contribution of this review is that it clearly maps out the direction of the impact (ie, positive or negative) of a number of indicators on quality judgment and the underlying reasons (ie, criteria) for the impact.

### Practical Implications

The identification and differentiation of positive and negative indicators provide clear guidance for online health information designers. They can incorporate positive indicators (eg, offering authors’ credentials and presenting information in a clear and organized way) and avoid negative indicators (eg, dead links and flash media format) to offer users better information seeking experiences. The fact that the same indicator (eg, government institutions as the source owner) can lead to different quality judgment for different people suggests that designers should also carefully investigate target users’ values and the corresponding criteria that they use to evaluate health information. This calls for active user research and user involvement in the design process.

The results of the review also have implications for consumer education. The review revealed a wide range of criteria that consumers use to evaluate the quality of online health information. Many of the criteria, such as familiarity, identification, relevance, practicality, and usefulness, are highly subjective and situational, influenced by factors such as information needs, online information search experience, and personal beliefs. In some cases, consumers assign such criteria higher priority than more objective ones such as expertise [57]. The review also revealed that consumers use a diverse set of quality indicators. The implications of some of the indicators are not well understood. For example, some consumers believe that the appearance of copyright information or the word “clinical” indicates high information quality [61,67]. Some consumers view the fact that a website passes the screening of virus/security software as an indicator of high quality [57]. There are also consumers assuming that third-party accreditations are indicators of information accuracy, when, in fact, the guidelines that these accreditations follow do not really check for information accuracy [74,75]. Consumers need education to use more objective criteria to evaluate online health information and understand the implications of a number of quality indicators.

### Limitations

This review has several limitations. First, we selected only studies where consumers explicitly described their quality evaluation behavior. These studies tend not to ask consumers to rate criteria or indicators; thus, we could not identify the importance of each indicator or criterion in quality judgment. Future reviews are needed to fill this gap. Second, we did not differentiate and compare results based on observations and results drawn from verbal inquiries as few included studies did. Eysenbach and Kohler [30] reported discrepancies between participants’ verbal accounts of what they do to evaluate health information and what they actually did in performing search tasks (based on observations). Thus, future empirical studies are needed to shed light on this gap. Third, in the coding process, we used criterion and indicator terms from the original papers, where feasible. In cases where we needed to infer criteria from indicators, we followed the mostly commonly recognized categorization by referring to prior empirical research and reviews or inferred the criteria from participants’ quotes. However, due to the different perspectives of the authors of the original papers and the inherent overlap between terms, such as comprehensiveness and completeness, our syntheses are inevitably affected by a certain degree of subjectivity. Fourth, because most studies treated the internet as one source of information without differentiating source types (eg, regular websites and social media), we were not able to identify whether the use of evaluation criteria and indicators differs by source type.

### Conclusions

The quality of online health information is a complex concept involving more than two dozen dimensions, as perceived by consumers. Although a set of core dimensions can be identified, the diversity involved in consumers’ use of criteria is too obvious to ignore. Further examination of contextual factors (eg, different source and user characteristics) that influence consumers’ application of quality criteria will bring further clarity to the concept. The review identified 165 indicators, to which criteria are applied to reach a quality judgment. Indicators could be source, content, or design related; they can have a positive or negative impact on quality judgment, contingent on situations and users’ values and beliefs. The identification and differentiation of positive and negative indicators along with their respective criteria can provide clearer guidance for designers of online health websites and educational interventions. Compared to experts’ evaluation, consumers’ evaluation of online health information relies heavily on peripheral cues and is influenced by various contextual factors (eg, personal beliefs and information needs). This finding suggests that current quality evaluation checklists, which are mostly based on experts’ view of quality, may not effectively serve the needs of consumers. Consumer behavior needs to be considered in the design of interventions that intend to promote quality evaluation in online searches. At the same time, it is worth noting that criteria and indicators used by consumers merit critical evaluation, as some criteria are overly subjective and the implications of some indicators are not well understood. User education is needed to address user misconceptions and the associated suboptimal evaluation behavior.

## Abbreviations

CDC: Centers for Disease Control and Prevention
CINAHL: Cumulative Index to Nursing and Allied Health
NIH: National Institutes of Health
